# Supplementary material for: Design and performance investigation of metamaterial-inspired dual band antenna for WBAN applications
Source: PLoS One. 2024 Aug 9;19(8):e0306737. doi: 10.1371/journal.pone.0306737 (PMC11315293; doi:10.1371/journal.pone.0306737)
Supplement: Attachment — Submitted filename: renamed_73759.docx [file pone.0306737.s001.docx]

**Authors Response to Reviewer’s Comments**

| **Reviewer-1** | |
| --- | --- |
| **Reviewer #1:** From the perspective of research, the authors' work is quite significant and important. My observations are as follows: | |
| **Comments** | **Author’s Response** |
| (a) The abstract contains the proposed name, objective, and research findings. | Thank you for very much for the valuable comment regarding the abstract of our paper. We acknowledge that the abstract contains the proposed name of the antenna, the objective, and the research findings. The objective of our work is to design and fabricate both the antenna and the metamaterial structure, followed by performance analysis in both on- and off-body scenarios. The research findings include achieving acceptable gain, adequate bandwidth, good radiation efficiency, and ensuring that the Specific Absorption Rate (SAR) value is below the FCC-specified threshold of 1.6 W/kg for any 1g of human body tissues.  Thank you again. |
| (b) The introduction contains the problem statement and the contribution of the work. | The authors of the manuscript are thankful for the comments provided by the honorable reviewer regarding the introduction of the manuscript. The authors acknowledge that the introduction contains the problem statement and outlines the contribution of the work.  The problem statement addressed in the proposed work pertains to the performance degradation and higher Specific Absorption Rate (SAR) associated with wearable antennas due to side and back lobe radiation, as well as frequency detuning caused by bending of the antenna. To mitigate these issues, metamaterials are being used as a promising solution.  The significance of the proposed integrated design, as summarized in the introduction section, lies in its ability to enhance performance in terms of gain, efficiency, and bandwidth when placed in proximity to the human body. Additionally, it ensures compliance with FCC-defined SAR safety limits. Furthermore, the proposed antenna design demonstrates stable performance despite various bending deformations.  Furthermore, the performance of the proposed work has been compared with similar recent literature, as presented in **Table 8**.  Thank you again. |
| (c) The dataset's information is properly cited or well explained, along with justification for the proposed antenna design and suitable outcomes of it. | Thank you for the comment regarding the dataset information in the proposed work. The authors appreciate your attention to detail. However, it is pertinent to clarify that no dataset is involved or required for the proposed work. The proposed study primarily focuses on the numerical simulation of the antenna and AMC designs. The simulation results are followed by fabrication and testing, with the outcomes presented and discussed in detail in the Results and Discussion section. We have ensured that the proposed antenna design and its outcomes are well-justified and adequately explained throughout the paper.  Thank you very much. |
| (d) The proposed outcomes should be compared with existing similar approaches, and the results are presented well in the form of graphs and tables. In this regard, a comparison table is expected. In addition, the authors had to briefly give insights into their work in the form of highlights. | Thank you for the valuable feedback. The authors would like to ensure that the outcomes of the proposed work have been compared with existing similar approaches, and the results are comprehensively presented in **Table 8**. Additionally, we have provided insights into our work in the form of highlights in **Section 5**, following the completion of the SAR discussion. We believe that these highlights effectively summarize the superior performance of the proposed work compared to existing literature.  Once again, thank you for the thoughtful review of the manuscript. |
| (e) The conclusion contains the findings of the work and the future scope.  The rest of it looks good, in my opinion | The authors of the manuscript are thankful for the valuable feedback and encouragement, and for acknowledging the content of the conclusion section. In the conclusion section, the findings have been summarized, including the achieved milestones in terms of gain, efficiency, bandwidth, and SAR. For further details on these findings, please refer to **Section 5** of the manuscript.  Additionally, the future scope of the proposed work has been extended to highlight potential areas for further research and development. The authors believe that these future directions will contribute to advancing the field and addressing any remaining challenges. The authors of the manuscript are very thankful for the encouragement and believe that this addresses the query of the honorable reviewer, providing clarity on the content of the conclusion section.  Thank you again. |
| **Reviewer-2** | |
| **Reviewer #2:** The authors have reported the work title “Design and Performance Investigation of Metamaterial-Inspired Dual Band Antenna for WBAN Applications,”. However, there are few queries which need to be addressed by the authors and are given below | |
| **Comments** | **Author’s Response** |
| Q1. Why Rogers RTDuroid5880 Substrate is termed as semi-flexible in abstract? (Rev-2) | The authors of the manuscript are thankful for the valuable feedback from the honorable reviewer.  The term 'semi-flexible' in the context of Rogers RT/Duroid 5880 substrate refers to its mechanical properties, specifically its ability to bend or fold to a certain extent without breaking or substantially deforming. Rogers RT/Duroid 5880 materials are available in various thicknesses, such as 1.6 mm, 0.57 mm, and 0.254 mm. The material with a thickness of 1.6 mm is rigid and cannot be easily bent or folded. However, RT/Duroid 5880 with thicknesses of 0.57 mm and 0.254 mm are flexible and can be bent or flexed to some degree without causing damage to the substrate or compromising its electrical properties. Nevertheless, it is not as flexible as some other materials like polyimide films, paper, polyester film, latex, or other textile/wearable materials, which are highly flexible. Therefore, it is termed as semi-flexible.  Thank you very much again. |
| Q2. What do you mean by in-phase characterization? (Rev-2) | Thank you very much for the valuable comment.  In-phase characterization refers to the analysis of the phase relationship between incident (plane wave) and reflected electromagnetic waves when interacting with the surface. Simply put, it involves investigating how the reflected wave aligns with the incident wave in terms of their phases. On the other hand, the surface reflection coefficient must be +1 (in-phase reflection) at the specified frequency, contrary to the traditional metal ground plane, which has a reflection coefficient of -1 (out-of-phase reflection). As we know, the perfect electrical conductor (PEC) reflects plane wave’s incident normal to its plane with a 180-degree phase shift, while the perfect magnetic conductor (PMC) reflects them with a 0-degree phase shift. So, the in-phase characteristic causes the metamaterial to act like a PMC [1-4].  In the context of the proposed work, characterizing the metamaterial in terms of in-phase reflection means evaluating whether the reflected wave maintains the same phase as the incident wave or if there is a phase shift introduced by the surface. It can be seen from the reflection phase of the proposed AMC structure (**Fig 7** in the manuscript) that within the desired bandwidth, the reflection phase exhibits variation from +90 degrees to -90 degrees, crossing the 0-degree reflection phase at the 2.45 and 5.8 GHz frequency bands. Within this bandwidth, the incident and reflected waves are in phase with each other.  Thank you again.   [1] Ullah, S. (2010). *Design and measurement of metamaterials based antennas* (Doctoral dissertation, Loughborough University). [2] Sievenpiper, D. F. (1999). *High-impedance electromagnetic surfaces*. University of California, Los Angeles. [3] Wang, C., Zhang, L., Wu, S., Huang, S., Liu, C., & Wu, X. (2021). A dual-band monopole antenna with EBG for wearable wireless body area networks. *The Applied Computational Electromagnetics Society Journal (ACES)*, 48-54. [4] Tamrakar, M., & Kommuri, U. K. (2021). EBG–AMC–HIS characteristics analysis of QBTR unitcell. *Sādhanā*, *46*, 1-6. |
| Q3. Add more analysis on AMC | The authors of the manuscript are thankful for the critical review.  In response to the honorable reviewer's suggestions for additional analysis on the AMC, the authors acknowledge the importance of comprehensive characterization. In our study, we have also conducted surface wave suppression characterization of the AMC surface using the suspended strip line method, as depicted in **Figure 1** below. However, the findings revealed that the surface did not provide surface wave suppression at the desired frequency. Instead, it exhibited in-phase reflection behavior. Considering the scope and length constraints of the manuscript, the surface wave suppression characterization of the proposed surface was not included in the main manuscript.  Thank you very much.    (a)    (b)  **Figure 1**. Surface wave suppression characterization (a) Simulation setup using suspended microstrip line (b) reflection (*S_11_*) and transmission (*S_12_*) coefficient of the proposed metamaterial surface |
| Q4. Give more insight and clear image on surface current density distribution on the surface? (Rev-2) | The authors of the manuscript are grateful to the reviewer for their valuable comment.  In response to the valuable suggestion/recommendation of the honorable reviewer, we have replaced **Fig 4** and **Fig 8** in the revised manuscript with clear images, as shown below. These images illustrate the distribution of surface current density on both the proposed antenna and the metamaterial structure.  Moreover, in the revised manuscript we have elaborated further on how electric currents are distributed across the surface of the antenna as well as the metamaterial structure.  We believe that these revisions provide more information and clarity regarding the surface current density distribution, as recommended.    **Fig 4.** Surface currents density of the proposed antenna at (a) 2.45 GHz (b) 5.80 GHz.    **Fig 8.** Surface currents density of the proposed AMC unit cell at (a) 2.45 GHz (b) 5.80 GHz.  Thank you again. |
| Q5. The slight deviation in S11 for bending analysis is observed. Give reasons. (Rev-2) | Thank you for the valuable feedback. The slight deviation in *S_11_* when analyzing the antenna in a bending scenario can be attributed to various factors, such as changes in geometry, mechanical stress, and alterations in the properties of the substrate material used. When the antenna bends, its geometry undergoes changes, which in turn affect its electrical properties, consequently impacting the propagation of electromagnetic waves. This variation leads to changes in impedance matching and, subsequently, the reflection coefficient. Additionally, bending imposes mechanical stress on the antenna structure, causing deformation in the material used. This stress can influence resonance frequency and impedance, thereby affecting the reflection coefficient. Furthermore, bending can cause the substrate to respond differently, affecting its permittivity. Consequently, changes in permittivity due to bending can also alter impedance matching and, thus, reflection characteristics.  Thank you very much again. |
| Q6. Why there are changes in S11 result when distance between antenna and AMC is varied? | Thank you for the valuable comments. Several factors can be attributed to the variation in the *S_11_* results when the distance between the antenna and the AMC varies. The distance between the antenna and the AMC plane affects the impedance matching of the antenna system. As the distance varies, the coupling between the antenna and the AMC plane alters the impedance seen by the antenna. This change in impedance leads to variations in the *S_11_* parameter. Additionally, when the antenna is placed in close proximity to the AMC plane, the near-field interaction between them is affected. Changing the distance between the AMC and antenna alters the electromagnetic field distribution near the antenna and the AMC plane, resulting in variations in the impedance seen by the antenna. Consequently, the *S_11_* is influenced.  Thank you very much again. |
| Q7. Why foam is preferred between Antenna and AMC? | The authors of the manuscript are grateful to the reviewer for their thoughtful review.  The foam material is preferably used because it serves to provide a smooth transition between the antenna and the AMC by having a permittivity equal to that of free space. This helps in achieving better impedance matching between the antenna and the surrounding environment. Additionally, the foam material acts as a buffer layer, which helps reduce reflections at the interface between the antenna and the AMC, leading to impedance matching and enhancing the overall antenna performance. Furthermore, foam is lightweight, flexible, and comfortable to wear, making it suitable for wearable applications. Moreover, it is easily available commercially in various thicknesses, making it convenient for prototyping and fabrication purposes. Its affordability compared to other specialized materials further contributes to its preference in wearable antenna designs.  Thank you. |
| Q8. Can you add mathematical modelling of AMC? | Thank you very much for the valuable comment and suggestions.  For the calculation of the length and width of the AMC, the well-known design equations proposed by D. Seivenpiper are adopted [1] [2]: This represents a common design approach involving the use of lumped *LC* elements (inductors and capacitors) to mimic the desired effective electromagnetic properties.  $C_{A}=\frac{W\varepsilon_{0\left( \boldsymbol{1}+\boldsymbol{\varepsilon}_{\boldsymbol{r}} \right)}}{\pi}\cosh^{-1} \left( \frac{W+g}{g} \right)$. (1)  $L_{s}=\mu_{0}h_{s}.$ (2)  These lumped elements are typically distributed along the structure to achieve the desired frequency response. Additionally, these design equations provide a starting point for designing a hexagonal AMC, but the actual design process may involve numerical simulations and optimizations to achieve the desired performance characteristics.  1. **DOI:** [10.1109/ACCESS.2018.2883379](https://doi.org/10.1109/ACCESS.2018.2883379)  **2.** DOI: 10.2528/PIER12060702  Thank you very much for the feedback. |
| Q9. Add back-lobe reduction technique comparison in Table 8. | Thank you very much for the valuable feedback and recommendation. A column has been added to **Table 8** in the revised manuscript to illustrate the back lobe reduction techniques proposed in the cited literature. |
| **Reviewer-3** | |
| **Reviewer #3:** Dear authors; | |
| **Comments** | **Author’s Response** |
| 1-Can you summarize in a few sentences what all this work is about at the end of the introduction? | The authors of the manuscript are grateful to the reviewer for their valuable comments.  Following the suggestions/recommendations of the honorable reviewer, a few sentences have been added at the end of the introduction section to summarize the main focus and achievements of the proposed work. |
| 2-The abstract needs more interest and rewriting of some paragraphs. | Thank you for your valuable feedback and suggestions regarding the abstract of the manuscript. We have carefully revised the abstract based on your recommendations to make it more engaging and coherent. Additionally, any inconsistencies in the paragraphs have been addressed.  The authors believe that the revised abstract now better highlights the significance of the proposed work and its contributions to the field.  Thank you once again for your critical review of the manuscript. |
| 3-There are still some aspects that can be improved (for grammar and punctuation). Improve the technical writing of your paper, where there are several grammatical errors and spelling, I think they need to be checked out. | Thank you for the thorough review and valuable remarks regarding the English language in the manuscript, which helped to ensure the quality of the proposed work. The entire manuscript has been carefully reviewed and checked for any potential English-language errors. To address this concern, we have used various tools, including professional proofreading services. Therefore, we believe that the manuscript is now free from any significant language issues.  Once again, the authors of the manuscript are thankful for thoroughly evaluating the manuscript and providing valuable comments. |
| 4-The conclusion needs more effort to elaborate on the achieved results with respect to future work, | Thank you for your valuable feedback. As per the suggestions/recommendations, the conclusion has been carefully revised in the revised manuscript to ensure that the results are thoroughly elaborated upon in the context of future work. In the revised conclusion, we have provided a detailed discussion on how the obtained results contribute to the existing body of knowledge in the field.  Furthermore, we have outlined specific areas for future research and identified potential avenues for further exploration based on the outcomes of our study.  Once again, thank you very much for the valuable feedback, which has helped us improve the quality of the work. |
| 5-There are still some aspects regarding the obtained results discussions that are missing. Can you please address your achievements well? | Thank you for the valuable comments. The comments have been taken into consideration to improve the clarity and comprehensiveness of the discussion on the achievements of the proposed work. The achievements of the proposed work are properly addressed in the results and discussion section. To ensure clarity and transparency in the findings of the proposed work, detailed explanations of the achievements, including gain, efficiency, bandwidth, and SAR, have been provided.  Furthermore, the findings/achievements of the proposed work have been compared with similar studies reported in the existing literature, as reflected in **Table 8**, which further signifies the contributions of the proposed work in relation to prior research efforts.  Thank you very much. |
| 6-The practical part is very important; therefore, I’m asking about the application specifications and research gap, | The authors are thankful the honorable reviewer for the thoughtful comments.  Regarding the practical aspects, the proposed work targets the application of health monitoring systems within Wireless Body Area Networks (WBANs), operating at 2.45 and 5.8 GHz frequency bands. The critical antenna parameters such as gain, bandwidth, size, efficiency, and Specific Absorption Rate (SAR) are meticulously considered. For short-range communication within a WBAN, a gain of around 2-3 dBi is necessary.  Additionally, the antenna must support the desired frequency bands with adequate bandwidth to accommodate signal variations and interference. Therefore, a bandwidth of at least 100 MHz or more is typically required to ensure reliable communication in dynamic environments. Higher antenna efficiency is crucial to minimize power consumption and maximize the operating range of WBAN devices. Moreover, the antenna's size is a critical factor for body-worn applications, with a size of a few square centimeters being desirable for integration into wearable gadgets. Furthermore, maintaining SAR values below the specified thresholds by regulatory bodies such as the FCC and ICNIRP is essential to protect human body tissues from harmful radiation.  The proposed antennas in our work meet these criteria, offering desirable gain (>6.6 dBi), adequate bandwidth (>7.4%), and high on-body efficiency (>84%) with SAR values (<0.71) well below the FCC threshold (1 W/Kg for any 1g of tissue mass) in both frequency bands. Additionally, the antenna's compact size makes it suitable for the proposed applications, as summarized in **Table 7 and 8**.  While the proposed work addresses most of the desirable features needed for the specified applications, there are still research gaps to be explored. These include further miniaturization, increasing bandwidth, optimizing efficiency, addressing SAR further, and exploring novel materials and fabrication techniques. By addressing these gaps, we believe we can further advance the field of wearable antennas for health monitoring systems, contributing to the development of more efficient, compact, and safe wearable devices for improved healthcare applications.  Thank you very much again for the valuable comments and feedback. |
| 7- The proposed topic is interesting, However, minor variations on the investigated subject have been noted. | The authors of the manuscript are thankful for the valuable feedback and for acknowledging interest in the proposed topic. We appreciate your attention to detail in noting minor variations on the investigated subject. While we agree with the honorable reviewer's comment that there may be some overlap with existing literature or minor variations on the topic, we believe that our study offers valuable contributions in several key areas. For instance, the compact size of the antenna meets the key requirement of wearable technology, while the incorporation of metamaterial significantly reduces SAR and increases antenna gain in both frequency bands. Additionally, our proposed design demonstrates a stable response under various bending conditions, even at severe degrees of bending. Furthermore, the design approach adopted in our work is simple and cost-effective. We are confident that our research adds depth and insight to the existing body of literature on this topic.  Thank you once again for your thoughtful review. |
| 8- The research method is not clearly described and is only partially appropriate for addressing the identified problem. | Thank you for your comments. We appreciate your attention to detail regarding the research methodology employed in the proposed work. We understand the concern and would like to assure that the research method we have employed is carefully aligned with the problem statement outlined in the manuscript. The research method involves a detailed analysis of the proposed antenna with and without the integration of AMC in both on- and off-body states. This approach enables thorough performance evaluation of the antenna under realistic conditions.  Additionally, to address safety concerns, we conducted comprehensive SAR analysis to ensure compliance with regulatory standards.  We believe that the thoroughness of our analysis, coupled with the comprehensive SAR evaluation, demonstrates the appropriateness of our research method for addressing the identified problem.  Once again, thank you for your valuable feedback |
| 9- The circuit model is very important? | Thank you very much for the valuable comment. We agree that the circuit model is very important for understanding the behavior of the proposed designs. We have made an effort to demonstrate its significance. **Figure 1 (a)** shows the equivalent circuit model of the proposed antenna along with its corresponding reflection coefficient, which shows good coherence with the reflection coefficient extracted from CST, as illustrated in **Figure 1 (b)**. We appreciate the feedback of the honorable reviewer, and we plan to further elaborate on the circuit model and its implications in the extended version of the proposed work.  Thank you again for the valuable feedback.    **Figure 1**: (a) Antenna equivalent circuit diagram. (b) comparison of the reflection coefficient response delivered by both CST and ADS |
| 10- The problem statement is unclear, and the literature review is articulated unclearly. | Thank you for the feedback and valuable insights.  The suggestions have been taken positively and the introduction section has been revised extensively to provide a clearer problem statement. The authors believe that these revisions have significantly improved the clarity and coherence of the manuscript. We hope that the revised manuscript would be satisfactory and will address the concerns.  Thank you again. |
| 11- The Results and Discussion section requires extension. The findings presented require a detailed justification and careful comparative analysis with previously published works to show their relevance and contribution to the field. | Thank you for the valuable comments and recommendations. All sections in the Results and Discussion have been carefully reviewed, and detailed justifications have been provided where appropriate. Additionally, the findings of the proposed work have been compared with existing literature and summarized in **Table 8**, while the significance and contribution of the work have been highlighted.  Thank you once again for the valuable comments aimed at improving the quality of the proposed work. |
| 12- The future impact of this work is not clearly defined in the content, limiting the impact of this work. Future work is an important part of the conclusion. | Thank you for your valuable comments. We appreciate the feedback of the honorable reviewer and agree that discussing future work is an essential aspect of the conclusion. We have taken suggestions/recommendations into consideration and have added more future work to the conclusion section. We believe that this additional content will enhance the comprehensiveness of the proposed work.  Thank you again. |
| 13- The results are still not matured well. Can you clearly mention what your objectives clearly for each result you got during the discussion process? | Thank you for the thoughtful review and comments.  The authors of the manuscript would like to clarify that the main objectives of the proposed study is clearly defined in the manuscript. The aim is to design and investigate the on- and off-body performance of a dual-band antenna operating in the 2.45 and 5.8 GHz frequency bands for WBAN applications, both with and without the integration of metamaterial.  Keeping in view the main objectives of the proposed study, we have successfully achieved these objectives. Through our experimental design and analysis, we have thoroughly investigated the performance of the proposed antenna and metamaterial in various scenarios. The results presented in the manuscript demonstrate that we have attained the desirable features such as high gain, acceptable on-body efficiency, adequate bandwidth, and stable reflection coefficient, as outlined in **Section 3**. Additionally, a summary of these findings is provided in **Tables 5** and **7** for reference.  Furthermore, in **Section 4**, the SAR analysis has been concluded, adhering to the FCC standard thresholds of 1.6 W/kg for any 1 g of human body tissues. The proposed designs resulted in a low SAR value, meeting the thresholds defined by the FCC, thus rendering the proposed antenna suitable for body-worn applications.  The authors believe that the results are aligned with the stated objectives, and we have made every effort to ensure clarity and coherence throughout the manuscript.  Thank you once again. |
| 14- The references need to be written in the same format. | The authors are very thankful for the recommendation and feedback. We have double-checked the references and ensured that they are written in the same format. |
| 15- There are several relative works, I wish to discuss. Can you please suggest your enhancements and advancements (if you would) over such as the following: A- <https://doi.org/10.1049/iet-nde.2020.0013> B- <http://dx.doi.org/10.2528/PIERM20113008> C- <https://doi.org/10.1002/dac.5024> D- <https://doi.org/10.1002/mop.32067> E- <https://doi.org/10.1007/s11277-020-07646-y> F- <https://doi.org/10.1017/S1759078720000665> G- <https://doi.org/10.1016/j.aeue.2010.03.008>  I loved this work and I feel it is very good. I hope these comments will help you improve this work after a major revision. Regards | The authors of the manuscript are thankful to reviewer for suggesting the valuable research articles. These articles are particularly interesting and helpful, especially in the context of energy harvesting using wearable technology and metamaterials. Among these, in the proposed work, we have also cited the most relevant articles to our work in the revised manuscript at appropriate places and have extended our future work based on the knowledge gained from these articles. We believe that these approaches strengthen the foundation of our research and contribute to the advancement of the field.  Furthermore, we agree that the suggested articles are valuable resources. Therefore, we recommend these articles to students, friends, and colleagues who are currently conducting research in antennas and metamaterials/metasurfaces, to implement these innovative ideas in their own work. We appreciate the feedback, and the extended version of our proposed work will incorporate the enhancements and advancements discussed in the suggested articles.  Thank you once again. |

**Table 8.** **Comparative study of the proposed integrated antenna with the existing literature.**

| **Ref.** | **Antenna Size**  **(mm^3^)** | **Substrate/**  **Flexibility** | **Operating Frequency (GHz)** | **Back Lobe Reduction**  **Technique** | **Gain (dBi)** | **Operating Bandwidth (%age)** | **Peak SAR (W/Kg)** | | **Antenna**  **Topology** |
| --- | --- | --- | --- | --- | --- | --- | --- | --- | --- |
|  |  |  |  |  |  |  | **1 g** | **10 g** |  |
| [47] | (0.33×0.35×0.012)λ_o_ | Rogers/  Semi-flexible | 2.4/5.8 | -- | 3.74/  5.13 | 3.8/  5.2 | 0.95/  0.478 | 0.57/  0.13 | Planner Monopole |
| [48] | (0.81×0.81×0.026)λ_o_ | F4B/No | 2.45/5.8 | -- | 1.9/  5.9 | 2.57/  5.22 | 0.254/  0.074 | -- | Circular |
| [49] | (0.81×0.81×0.016)λ_o_ | Felt/ Yes | 2.45/5.8 | -- | 6.33/  6.98 | 4.9/  3.8 | 0.042/  0.09 | -- | Circular |
| [50] | (0.98×0.98×0.027)λ_o_ | Textile/Yes | 2.45/5.8 | AMC | 6.4/  7.6 | 4/12 | 0.08 | -- | Rec. Patch |
| [51] | (0.82×0.82×0.024)λ_o_ | Textile/ Yes | 2.45/5.5 | AMC | 2.5/  0~4 | 12/  16.3 | 0.019/  0.009 | -- | Rec. Patch |
| [52] | (0.67×0.67×0.016)λ_o_ | Felt/ Yes | 2.45/5.8 | EBG | 4.5/  5.6 | 4.2 | -- | 0.661/  1.51 | PIFA |
| [53] | (0.82×0.74×0.024)λ_o_ | Felt/ Yes | 2.4/5.8 | -- | 2.9/  5.0 | 6.54/  11.5 | -- | 0.056/  0.067 | SIW cavity antenna |
| [54] | (0.51×0.61×0.022)λ_o_ | Denim/ Yes | 2.45/5.8 | EBG | -- | 4.3/  5.57 | -- | 0.031/  0.040 | Monopole |
| **This Work** | (0.55×0.55×0.002)λ_o_ | Rogers/  Semi-flexible | 2.45/5.8 | AMC | 6.61/  8.02 | 10.12/  7.43 | 0.651/  0.711 | -- | Monopole |
| **λ_o_ is the free space wavelength at 2.45 GHz.* | | | | | | | | | |
